# The impact of care pathways for exacerbation of Chronic Obstructive Pulmonary Disease: rationale and design of a cluster randomized controlled trial

**DOI:** 10.1186/1745-6215-11-111

**Published:** 2010-11-19

**Authors:** Kris Vanhaecht, Walter Sermeus, Jan Peers, Cathy Lodewijckx, Svin Deneckere, Fabrizio Leigheb, Marc Decramer, Massimiliano Panella, the EQCP Study Group

**Affiliations:** 1 European Pathway Association, Kapucijnenvoer 35/4, B-3000 Leuven, Belgium; 2 Faculty of Medicine, Catholic University Leuven, Belgium; 3 Faculty of Medicine, Amedeo Avogadro University of Eastern Piedmont, Italy

## Abstract

**Background:**

Hospital treatment of chronic obstructive pulmonary disease (COPD) frequently does not follow published evidences. This lack of adherence can contribute to the high morbidity, mortality and readmissions rates. The European Quality of Care Pathway (EQCP) study on acute exacerbations of COPD (NTC00962468) is undertaken to determine how care pathways (CP) as complex intervention for hospital treatment of COPD affects care variability, adherence to evidence based key interventions and clinical outcomes.

**Methods:**

An international cluster Randomized Controlled Trial (cRCT) will be performed in Belgium, Italy, Ireland and Portugal. Based on the power analysis, a sample of 40 hospital teams and 398 patients will be included in the study. In the control arm of the study, usual care will be provided. The experimental teams will implement a CP as complex intervention which will include three active components: a formative evaluation of the quality and organization of care, a set of evidence based key interventions, and support on the development and implementation of the CP. The main outcome will be six-month readmission rate. As a secondary endpoint a set of clinical outcome and performance indicators (including care process evaluation and team functioning indicators) will be measured in both groups.

**Discussion:**

The EQCP study is the first international cRCT on care pathways. The design of the EQCP project is both a research study and a quality improvement project and will include a realistic evaluation framework including process analysis to further understand why and when CP can really work.

**Trial Registration number:**

NCT00962468

## Background

Healthcare is changing towards more patient focused care. The organization of the care process related to quality, efficiency and accessibility is one of the main areas of interest within the next years for clinicians, healthcare managers and policy makers. A main method to (re)organize a care process is the development and implementation of a care pathway. Care pathways, also known as clinical pathways or critical pathways, are used worldwide for a variety of patient groups [1-7]. A care pathway is defined as a complex intervention for the mutual decision making and organization of predictable care for a well-defined group of patients during a well defined period. Defining characteristics of pathways includes: an explicit statement of the goals and key elements of care based on evidence, best practice and patient expectations; the facilitations of the communication and coordination of roles, and sequencing the activities of the multidisciplinary care team, patients and their relatives; the documentation, monitoring, and evaluation of variances and outcomes; and the identification of relevant resources [4,8,9].

A care pathway is explicitly defined as a "complex intervention" [4,8-11]. Complex interventions in healthcare, whether therapeutic or preventive, comprise a number of interacting components which seem essential to the proper functioning of the intervention although the "active component'' of the intervention that is effective, is difficult to specify. Considering a spectrum of low to high complexity such as developing a drug would be at the low end of complexity spectrum and the evaluation of the effect of a stroke unit would be at the high end of the spectrum. The greater the difficulty in defining precisely what the "active component" of an intervention is, and how they relate to each other, the greater the likelihood that you are dealing with a complex intervention [12-14]. Pathways in the line of stroke units seem to be at the higher end of the complexity spectrum. Active ingredients of a care pathway complex intervention might be the level of multidisciplinary teamwork, the integration of a package of evidence based key interventions and the active follow-up of the care process [6].

A recent Cochrane review concludes that clinical pathways are associated with reduced in-hospital complications and improved documentation without negatively impacting on length of stay and hospital costs [15]. The effects are however still quite small in comparison of what we might expect theoretically. One possible reason is the high variability in effect from one organisation to another organisation what seems to stress that context factors might be extremely important [9,16,17]. These context factors are not taking well in these meta-analyses as context is really stripped off. An indicator of context influence is the heterogeneity of the meta-analysis, which seems to be quite high in the Rotter meta-analysis paper [15]. Large multicenter trials including information on the mechanisms used and the context of the involved organizations will be important to fully understand why and when pathways lead to their effect [9,18].

To evaluate pathway effectiveness the European Pathway Association, an international not for profit association, launched the European Quality of Care Pathways (EQCP)-study on exacerbation of Chronic Obstructive Pulmonary Disease (COPD) [18]. COPD is worldwide a leading cause of acute hospital admission [19]. Patients with COPD exacerbation need multidisciplinary care and the coordination of the care process among multiple caregivers is complex [20]. Thirty five percent of COPD patients are admitted because of acute exacerbation within one year [21]. Up to 40% of admitted patients having two or more readmissions a year [22,23]. A systematic review about in-hospital management of COPD exacerbation showed that implementation of care processes recommended by worldwide accepted guidelines is very poor and show high variance, especially for performance of non pharmacological interventions [24]. Also Decramer et al. (2003) found several important deviations from international guidelines in the management of COPD by pulmonologists and general practitioners [25].

Up to now two non-randomized trials about the impact of a care pathway for inpatient management of COPD exacerbation are published [26,27]. The studies have been conducted between 2000 and 2001, and the methodology was rather weak. However the studies suggest that a COPD exacerbation care pathway improves performance with regard to diagnostic assessment and use of standing orders, and that it may reduce length of stay and the number of hospitalisations.

### Objectives

The primary goal of the EQCP study on COPD is to evaluate the care pathway effectiveness in acute hospitals. A secondary goal is to understand how and under which circumstances the implementation of a pathway for COPD is successful.

## Methods

### The project

The European Quality of Care Pathways (EQCP) study is an international multicentre research project launched the European Pathway Association (E-P-A) http://www.E-P-A.org, an international not for profit association [18]. The E-P-A is collaborating with the Center for Health Services and Nursing Research of the Faculty of Medicine of the Catholic University Leuven (Belgium) and the School of Public Health of the Amedeo Avogadro University of Eastern Piedmont (Italy) for the scientific lead of the this study. The study runs in four countries: Belgium, Ireland, Italy and Portugal. In each country, a research centre is coordinating the project in the own country based on the international agreed protocol. In Belgium the lead coordinating centre is the Center for Health Services and Nursing Research of the Faculty of Medicine of the Catholic University Leuven. For Ireland, the lead centre is the Health Service Executive in Dublin. In Italy, the School of Public Health of the Amedeo Avogadro University of Eastern Piedmont is coordinating the project with support from ARESS Piemonte. In Portugal, the lead coordinating centre is National School of Public Health in Lisbon. In each of the four countries hospitals will be selected by E-P-A in close cooperation with a national coordinator. In every participating hospital a pathway facilitator is appointed as local facilitator for implementing the care pathway. The pathway facilitator is trained by the E-P-A team in implementing care pathways, except for Belgium where hospitals were selected among members of the Belgian-Dutch Clinical Pathway Network and all pathway facilitators were trained before in the network [18].

### Study design

To evaluate the effect of a care pathway, a cluster Randomized Controlled Trial (cRCT) will be used [12-14]. In cRCTs organizations, rather than individuals, are randomized to an intervention and a control group, and outcomes are measured on individual level within the clusters [14]. Each cluster consist of patients hospitalized for a COPD exacerbation in a particular hospital and cared for by a specific multidisciplinary team.

**Figure 1:**
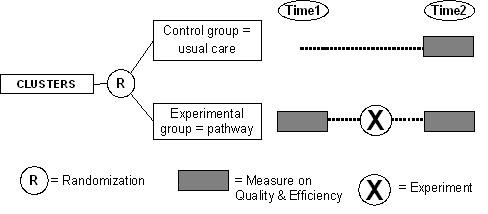
**The EQCP study design**.

Stratified randomization will be used to assign hospitals to an intervention group (development and implementation of an evidence based care pathway) and a control group (no intervention/usual care). Literature showed that several organizational factors could have significant impact on performance of key interventions and outcomes. To ensure that hospitals in both arms are in balance, hospitals will be stratified on country-level, according to the following variables: hospital type (teaching versus non teaching), hospitals size (< 600 and ≥600 beds) and annual volume of COPD-patients (< 300 and ≥300). Furthermore, the following organizational factors will be explored: availability of an early discharge scheme, presence of local guidelines about follow-up of patients after discharge, presence of a formal pulmonary rehabilitation program, availability of non-invasive ventilation, and annual volume of patients treated for COPD exacerbation. These variables will be treated as covariates in the data analysis. To the hospitals included in the control group will be offered the opportunity to develop a care pathway one year later, based on the protocol and experience gained in the experimental group, in order to increase their participation. By this means the design incorporated some characteristics of a step wedge design in which the implementation of the intervention is spread over time (see figure 1).

### Inclusion - exclusion criteria

Organizations will be included if they will provide written agreement to participate and agree that they will not develop and implement a pathway for COPD within the time frame of the study when randomized in the control group. All consecutive patients admitted for COPD exacerbation will be included in the study if (i) they will provide written informed consent; (ii) they will be hospitalized for at least 48 hours, (iii) they will be admitted on the ward where the COPD exacerbation is usually treated (respiratory ward, geriatric ward, general medicine ward) and (iv) if they will be able to understand and read the native language. Each patient will be included only once in the study at initial admission, even if the patient will be hospitalized more than once during the study period. Patients will be excluded from the study if they will require invasive positive pressure ventilation, or if they are already included in another study of which the measurements could influence the measurements or outcomes of the EQCP-study.

### Study sample

Sample size calculation in a cRCT is based on the improvement in the main outcome parameters [13,14]. The selection of main outcomes for the EQCP-study is based on three criteria: frequency of use in the literature, opinions of experts and timing of the research project with respect to organizational constraints (sustainability of the design, time to include patients). Based on these criteria, six-month readmission rate was identified as the main study outcome for in-hospital management of COPD exacerbation ([24]; Lodewijckx C, Sermeus W, Vanhaecht K, Panella M, Deneckere S, Leigheb F, Troosters T, Decramer M. Selection of indicators for research on COPD care pathways: an international Delphi study. submitted). Two other important outcome parameters are 1-year mortality and length of in-hospital stay. Based on a power of 80% and an α of 0.05 (two-sided), 296 patients per arm are needed to observe a 10 percent reduction rate in readmission of 41% to 30% [28,29]. After adjustment for the cluster design, based on two previous cRCT by Panella et al [30,31] (ICC: 0.018; IFF: 1.342; n = 20) the effective sample size increased to 398 patients per arm. This means that, based on a number of 20 consecutive admitted patients in each unit, 20 hospitals should be included both in each intervention and control groups. As four countries are actually involved in the study, every country has to enroll at least ten hospitals that have to be randomized into five cases and five controls [30,31].

### The complex intervention: care pathway implementation in the experimental arm

**Figure 2:**
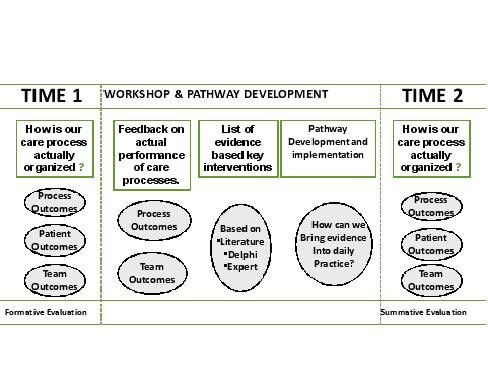
**The EQCP complex intervention**.

The care pathway will include three active components: (i) A formative evaluation on the quality and organization of the care process that will be performed by measuring performance of key interventions (see figure 2). These interventions are the most important interventions which have impact on the quality of care or the length of stay. They are performed by the medical doctors, nurses, physiotherapists and social workers. Key interventions are given in the domain of medication, the laboratory tests, patient information and discharge planning.

Furthermore a set of team indicators will be measured. Feedback will be provided on the data obtained to help the teams in understanding their bottlenecks and the actual overall organization of the care process. Therefore a pretest will be performed at 6 month before developing the care pathway during a 2-3 months period. During this pretest, usual care for 20 consecutive patients is measured against a set of key-interventions (see ii). All data will be transferred to the research center for analysis. A formative feedback report will be produced describing the relative performance of the team against the protocol and against the performance of all other teams in the study. (ii) A set of evidence based key interventions will be provided to the multidisciplinary team. This set is based on an extensive literature review, Map of Medicine^® ^http://www.mapofmedicine.com and on consensus by international clinical experts using a Delphi-survey ([18,24] Lodewijckx C, Sermeus W, Vanhaecht K, Panella M, Deneckere S, Leigheb F, Troosters T, Decramer M. Selection of indicators for research on COPD care pathways: an international Delphi study. submitted). The key interventions and outcomes include both in-hospital interventions and information for a safe discharge. (iii) The pathway facilitators will be supported to improve the organization of the care process by developing and implementing a care pathway, based on the findings of the evaluation of the care process and the set of evidence based key interventions. The pathway facilitators make use of a care pathway implementation protocol based on the Deming-PDSA cycle which is generally accepted as the standard method for quality improvement. Meetings with the pathway facilitators are organized to present and discuss the feedback report and to discuss actual bottlenecks in implementing the care pathway. Change will be supported by giving the possibility to exchange best practices among participants. During these meetings local clinical champions and team change experts will help and stimulate the pathway facilitators to effective knowledge sharing processes [18].

### The control group

In the control group the complex intervention will not be implemented and these teams will provide usual care. The control hospitals agree not to change their actual organization of the care process and do not develop a care pathway during the study period. The team members will provide care in the same way as they were doing before the start of the study.

### Measurements

To measure the effect of the care pathways on the process and outcome indicators, a set of process and outcome indicators to evaluate the effectiveness of in-hospital management of COPD exacerbation was developed based on literature [24]. Following literature sources were reviewed: (I) the guidelines of Global Initiative for Chronic Obstructive Pulmonary Disease [20]; (II) the guidelines of the American Thoracic Society-European Respiratory Society Task Force [32]; (III) an extensive review on management of COPD exacerbation published in 2006 by Rodriguez-Roisin [33]; (IV) the European Respiratory Monograph, a book on the management of COPD, published by the European Respiratory Society (ERS) in 2006 [34], (V) The guidelines of the National Institute for Clinical Excellence (NICE) (2004) [35], (VI) two process flows regarding management of COPD exacerbation in Map of Medicine [36] and (VII) a systematic review about in-hospital management of COPD exacerbation [24]. Additionally, three controlled trials about impact of COPD pathways or reviews of COPD and pathways [26,27,37], and 11 COPD outcome studies were reviewed [28,29,39-46]. Consent about the final set of indicators was obtained using an international Delphi study ([18]; Lodewijckx C, Sermeus W, Vanhaecht K, Panella M, Deneckere S, Leigheb F, Troosters T, Decramer M. Selection of indicators for research on COPD care pathways: an international Delphi study. submitted). These indicators were translated in concrete measurements by a multidisciplinary expert panel during a consensus meeting: a medical doctor, a physiotherapist, a clinical nurse specialist and the researchers of the EQCP-study [18].

To further understand why pathways work, information on the context of the organization is important. Within the EQCP study a set of both generic and COPD specific context indicators and team structure indicators will be developed based on literature review, an international Delphi study and expert opinion [18].

### Registration and Ethical approval

The study is registered as a cluster randomized clinical trial at clinicaltrials.gov (identifier: NCT00962468). The ethical approval will be country specific, but overall ethical approval will be sustained on three levels: (i) Ethical approval by the ethical committee of the coordinating centre on country level (ii) Ethical approval with regard to the participation in the intervention will be sought on cluster level, namely by the ethical committee of each of the participating hospitals. These committees can agree or disagree with the overall approval of the coordinating centre. As indicated by the Medical Research Council, patient's consent to participate in the study is not possible, because randomization occurred at the hospital level and not on patient level. Moreover the aim of the study is to improve adherence to evidence based care through clinical pathways in the intervention group. In the control group, no intervention will be implemented and patients thus will receive usual care. Therefore experimental as well as control group should not imply any risk for the patients included. (iii) Individual informed consent will be sought from the patient with regard to the access of the patient record and participation in surveys. At the present time the approval of the ethical committee of the coordinating centre at Leuven University was obtained (identifier: ML5617) and the proposal is submitted for approval in Portugal, Italy and Ireland.

## Discussion

The EQCP study is the first international cluster randomized controlled trial on the effect of care pathways [18]. Within this study a cRCT design is combined with a realistic evaluation approach [47]. In this way the differences between the experimental and control arms can be analyzed but also the process evaluations within the experimental arm itself can be followed-up and evaluated [48]. As suggested by Berwick in 2008 it is not only important to understand if an intervention works but why and under what circumstances it works [16]. The approach in the EQCP study will allow us to analyze if pathways work but also provide information on the when and how [18]. Within this international trial, three active ingredients define the complex intervention: the feedback on the actual situation, the information on the evidence based key interventions and the implementation and design process.

Hawe and colleagues argue that the crucial point in the evaluation of complex interventions lies in what is standardized and that in complex interventions, the function and process of the intervention should be standardized and not only the components themselves [49]. This argument is important for pathway research and was previously described by Panella et al [10]. Rather than defining the components of the intervention as standard, what should be defined as standard are the steps in the change process that the elements are purporting to facilitate or the key functions that they are meant to have [49]. In pathway research the pathway process or quality improvement cycle that is run through is part of the intervention. In that way the improvement and implementation process is included as one of the basic active ingredients. One challenge in multicenter trials on pathways, or quality improvement methods in general, or in comparing pathways from or between different organizations is to understand the context. Pawson & Tilley define that an action is causal only if its outcome is triggered by a mechanism acting in a context (context + mechanism = outcome) [47]. They argue that programs work (so have good outcomes) only insofar as they introduce the appropriate ideas and opportunities (the mechanism) to groups in the appropriate social and cultural conditions (the context) [16,47]. This realistic evaluation paradigm has already been used in pathway research [4] and was recently suggested by Berwick on the science of improvement [16]. For pathways the mechanism will need to be based on the basic active ingredients as described above but the fine-tuning of the intervention will be based on the actual bottlenecks and on the context of the organization and multidisciplinary team involved. Therefore in the actual international cRCT on pathway also a set of team indicators and organizational factors are measured. The process and outcome indicators will provide data to understand if pathways work, but the team indicators will be of help in understanding why and how they work.

With this study E-P-A will be able to influence health professionals and hospital managers in actively improving the quality and efficiency of care [18]. The teams receive support in the re-organization of the COPD care processes and can later use this implementation knowledge in other care processes. Teams will receive feedback on their actual organization including benchmark data with other international teams. In this way the design of the EQCP project is both a research study and a quality improvement project.

## Abbreviations

ARESS: agenzia per i servizi sanitari regionali; COPD: chronic obstructive pulmonary disease; CP: care pathway; cRCT: cluster randomized controlled trial; E-P-A: European Pathway Association; EQCP: European Quality of Care Pathway; GOLD: Global Initiative for Chronic Obstructive Lung Disease; PDSA: plan do study act

## Declaration of competing interests

The authors declare that they have no competing interests.

## Authors' contributions

KV, WS, CL, SD, MP contributed to the draft and final version of the paper. JP, FL and MD have been involved in the setup of the study. JP is chairman of the steering committee of the EQCP research group. MD supervised the selection of the clinical indicators. MP, KV and WS have the scientific lead of the study. KV is international coordinator of the study. All members of the EQCP Study Group have been involved in the organization of the study in the four participating countries. All authors have read and approved the final manuscript.
